# A Mobile Application-Based Relational Agent as a Health Professional for COVID-19 Patients: Design, Approach, and Implications

**DOI:** 10.3390/ijerph192113794

**Published:** 2022-10-24

**Authors:** Beenish Moalla Chaudhry, Ashraful Islam

**Affiliations:** School of Computing and Informatics, University of Louisiana at Lafayette, 301 East Lewis Street, Lafayette, LA 70503, USA

**Keywords:** older adults, goal-oriented care, connected health, mobile application, human-centered design

## Abstract

Relational Agents’ (RAs) ability to maintain socio-emotional relationships with users can be an asset to COVID-19 patients. The goal of this research was to identify principles for designing an RA that can act as a health professional for a COVID-19 patient. We first identified tasks that such an RA can provide by interviewing 33 individuals, who had recovered from COVID-19. The transcribed interviews were analyzed using qualitative thematic analysis. Based on the findings, four sets of hypothetical conversations were handcrafted to illustrate how the proposed RA will execute the identified tasks. These conversations were then evaluated by 43 healthcare professionals in a qualitative study. Thematic analysis was again used to identify characteristics that would be suitable for the proposed RA. The results suggest that the RA must: model clinical protocols; incorporate evidence-based interventions; inform, educate, and remind patients; build trusting relationships, and support their socio-emotional needs. The findings have implications for designing RAs for other healthcare contexts beyond the pandemic.

## 1. Introduction

The COVID-19 pandemic not only strained and disrupted the normal operations of healthcare facilities [1] but also posed considerable burden on the healthcare professionals (HCPs). Research indicated that increased hours and responsibilities, and other concomitant COVID-19 stressors, caused exhaustion, mental fatigue, and post-traumatic stress disorder (PTSD) among the healthcare workers [2,3]. Several hospitals experienced staff, specifically nurse, shortages, which directly impacted patient safety and quality of care [4,5]. Thousands of healthcare workers lost their lives due to exposure and lack of personal protective gear [6]. On the other hand, patients avoided or delayed hospital visits for routine and critical healthcare, which, in many cases, caused deterioration of patient conditions or put them at risk of complications [7,8]. It has been anticipated that widespread delayed care and post-COVID-19 health conditions will result in higher hospitalization rates and need for more complex hospital care in the future [9,10], which is a current concern for hospitals. The mental and behavioral health issues have also increased due to the pandemic, posing post-COVID-19 challenges for the healthcare systems [11].

### 1.1. Background and Motivation

In the light of these challenges, it is imperative to develop and employ alternate strategies to deliver patient care and to ensure healthcare worker safety. This can be achieved by keeping the COVID-19 patients, who do not need to be hospitalized, out of the hospital, and delivering healthcare services outside of the traditional care settings. Telehealth [12] which allows providers and patients to interact remotely, without risking infection, garnered considerable attention during the COVID-19 pandemic [13]. However, there are logistical challenges associated with delivering care via this channel [14]. Telehealth may be unsuitable for patients who lack appropriate devices, or do not have the Internet access for video calls. Telehealth tools are often not integrated within providers’ workflows. Limited or lack of reimbursement hinders its widespread adoption of telehealth [15]. Its usefulness is also constrained by providers’ availability; for example, a suitable provider may be unavailable at night or during an emergency [16].

For over a decade, researchers have been interested in developing Relational Agents (RAs) that can act as provider substitutes and remotely deliver various healthcare services [17]. RAs are described as virtual agents (VAs) or computational artifacts that are tasked with the responsibility of maintaining virtual socio-emotional relationships with their users over a protected period [18]. Earlier research has shown that VAs can build rapport with patients that can be useful in screening patients for various diseases, especially those that are stigmatized [19,20]; facilitating self-management by educating, advising, and helping patients adopt and maintain healthy habits [21]; and promoting mental health through counseling and therapy [22,23]. VAs can overcome many shortcomings of traditional telehealth, including the potential unavailability of HCPs in time of crisis — unlike HCPs, VAs are always available on users’ mobile devices. Moreover, VAs can provide various health services at minimum cost.

During the COVID-19 crisis, a proliferation of mobile device-based VAs emerged on the healthcare landscape [24]. A number of VAs were developed to provide psychological support during the post-quarantine periods [25,26] and to increase infection awareness among individuals [27,28]. While these works explore VAs’ feasibility in delivering essential COVID-19 related health services, none investigates the true potential of RAs, i.e., their ability to maintain sustained socio-emotional relationships with patients and deliver health services according to patients’ unfolding needs. This is a missed opportunity because these RA qualities can be extremely beneficial to COVID-19 patients as they face self-management burden while contending with disease progression and uncertainty in self-isolated circumstances, characterized by minimal interaction with traditional healthcare facilities and caregivers.

Inspired by this research gap, the objective of this research was requirements analysis, i.e., determination of the needs and expectations of patients and healthcare professionals for an RA that aimed at providing COVID-19 related health services. Understanding what users want and expect from a technical product before it is developed is important for the success of the product. If a product is found to be incompatible with users’ expectations after it has been developed, then it will be abandoned by the users and resources invested in its development will be wasted. Therefore, requirements analysis research is an essential first step when a new technical product is being planned.

### 1.2. Literature Review

#### 1.2.1. Conversational Agents

A VA is a computational dialogue system that not only understands human conversations but also responds in natural language. The most basic type of a VA is a conversational agent (CA) that is usually programmed to perform specific tasks using written or spoken natural language, and utilizing simple rule-based technique, or artificial intelligence [29]. Rule-based CAs work by matching a predetermined set of topics with predefined answers, whereas artificial intelligence-based CAs can expand on their functionalities using machine learning and natural language processing to understand user queries and engage in complex conversations. The first CA ELIZA [30] was a rule-based system developed by Weizenbaum in 1966 to provide patient-centered psychotherapy.

The earlier CA research was more concerned with producing human-like natural language; these days researchers examine how other communicative behaviors, such as physical body, gestures, humor, and communication style, can be utilized to communicate with humans [31,32,33,34]. This has given rise to ‘Embodied conversational agent’ (ECA) that use physical representations, particularly a body and, sometimes, also gestures, to facilitate interaction between a person and a computer. RAs are VAs that focus on maintaining long-term relationships with their users. They may use a physical form (embodiment), and both verbal and nonverbal (e.g., gestures, eye gazes) representations to develop and sustain deep, and meaningful connections with their users over an extended period [35]. The first RA called Laura was introduced by Bickmore et al. [36], whose goal was to inspire users to engage in physical activity, by interacting with them on a regular basis, recalling previous interactions, and responding to user’s activities with empathy. The acceptance study of Laura indicated that users found it easy to use, and they were satisfied in terms of relationship, trust, friendliness, and information it provided. These findings paved the way for the researchers to explore applications of RAs in other healthcare domains.

#### 1.2.2. VAs for Screening

Lucas et al. created a VA with human-like appearance, characteristics, and abilities (virtual human or VH) to screen military personnel for PTSD symptoms [19]. The VH used a conversational style to ask a series of questions that are the same as those asked by a healthcare professional in face-to-face clinical settings. The system was tested by 156 target users over two stages, and the results showed that participants were more willing to disclose their PTSD symptoms to a VH interviewer than on official and anonymized versions of paper-based PTSD assessments. The researchers concluded that VHs could evoke emotional reactions in patients and build trusting relationship with them, which may not be possible in real life situations and clinical settings.

Zalake et al. [20] created an Internet-based VH to aid self-screening of colorectal cancer. For greater adaptability, this VH was customized to the user’s gender and race, and it used a conversational style to conduct screening. The design of the conversational prompts considered ten factors including disease severity, response efficacy, risk probability, constraints, and narrative persuasion with the goal of removing screening obstacles such as a lack of doctor’s advice and a lack of patient awareness. The prompts were iteratively refined with feedback from the potential users. The users also reported that the personalized (according to user’s demographics) appearance of the VH had a profound effect on them, and the conversational prompts encouraged them to explore varied health options.

Overall, the existing work suggests that VH interviewers provide a safe, reduced-stigma environment and they can build rapport with users that encourages them to disclose sensitive information for diagnosis of health conditions. Moreover, both verbal (e.g., choice of words, intonation, back channel utterances) and non-verbal (e.g., appearance, facial expression, eye gazes, gestures, postures) behaviors of VH influence rapport building aspects with users that ultimately has an impact of user’s willingness to reveal information about themselves to the VH.

#### 1.2.3. VAs for Health Promotion

While there are various types of social supports, the most studied social support behaviors in human-agent interactions have been informational, emotional and esteem supports [37]. Informational support occurs when the agent shares knowledge or facts that can help users reflect on their existing actions and behaviors as well as learn new skills and behaviors. Emotional support occurs when agent makes utterances that express care, regard, compassion, and concern for the user. It is also manifested when the agent expresses praise and encouragement for user’s behavior. Esteem support focuses on promoting user’s skills, abilities, and intrinsic values. The purpose of these supports is to increase users’ belief in their ability to care for themselves, make commitments, overcome barriers, manage expectations, and ultimately maintain healthy behaviors.

When designing VAs for purposes of health promotion, researchers tend to focus on refining their social support behaviors. For example, Olafsson et al. [38] designed an ECA to provide emotional and informational social support to patients receiving medication-assisted treatment (MAT) for opioid use disorder. The emotional support was achieved through casual social chatting while informational support consisted of teaching patients two exercises commonly used in MATs, i.e., emotion recognition and mindfulness with deep breathing, followed by an interactive session focusing on emotion regulation. A pilot evaluation of the ECA showed that the participants found the agent interesting, convenient to work with, and reliable, which enhanced their eagerness to use the system. The same researchers, subsequently, designed two additional ECAs [21] to enable people to adopt healthy lifestyles, such as increasing physical activity or eating more fruits and vegetables. These ECAs employed the motivational interviewing technique during their conversations with the users to fulfill users’ esteem needs. In addition, the counseling conversations occasionally used humor to provide emotional support to the user. The ECAs had a substantial impact on participants’ desire to enhance their healthy behaviors, according to a user study. Another ECA called Greta provided two types of social support to adolescents to help them overcome their eating disorder. The informational support took the form of lessons about overcoming eating disorder and general knowledge about the disorder. The emotional support took the form of empathetic feedback that was provided when adolescents were frustrated with the coping challenges [39].

Overall, research has demonstrated the positive effects of VAs’ social support on their users’ health outcomes, irrespective of the social support type.

#### 1.2.4. VAs in Mental Health

Several researchers have highlighted the therapeutic efficacy of RAs in the treatment of mental health issues. Bott et al. [40] performed a clinical trial on 95 hospitalized individuals, including healthcare staff, to see how a human-in-the-loop protocol-driven RA affected them. This RA’s interactions were crafted with animal avatars and were developed based on a software-driven expert system and a team of health advocates. The health advocates were responsible for providing written responses based on user interaction and feedback through the non-human avatars. Interactions with the avatar were shown to alleviate participants’ mental stress, depression, loneliness, and falls during their hospital stay. Further evaluation of this RA with other populations has shown that VA interactions when controlled and monitored by humans can prevent development of mental health issues in a variety of populations.

Tielman et al. [41,42] have also made important contributions to designing CA providing post-therapy stress management strategies to patients recovering from PTSD at home. For PTSD treatment, they created a CA prototype and a motivational messaging system. The CA utilized an ontology supported method to interact with the users where the topics were hierarchically classified with sub-classes based on location, actions, people, emotions, objects, and senses. Depending on the user’s choices over a set of questions on the topics, the agent determines the appropriate one and moves forward accordingly. On the other hand, the motivational messaging system improves post-therapy stress by delivering messages to users from a database using a likelihood estimation method based on symptom trends and trust levels.

Fitzpatrick et al. [43] created ‘Woebot’, a fully automated web-based CA that provides cognitive behavioral therapy (CBT) to relieve anxiety and depression symptoms. Woebot provided CBT by constant mood monitoring and conversations through an instant messaging app. The conversational approach was influenced by human clinical decision-making and social discourse dynamics. Empathetic responses, personalized content, target settings, meditation, and interaction-feedback features were all included in the conversations to tackle the users’ mood during CBT sessions. Woebot was studied on 70 patients with anxiety and depressive symptoms, and it was shown to be effective in minimizing the severity of their symptoms in any case.

Gaffeney et al. [44] designed a web-based platform named ‘MYLO’ for cognitive therapy. MYLO employed textual conversations without any assistance from any HCPs. The conversations by MYLO were delivered based on various themes e.g., anger, control, conflict, perception, etc. and the themes were determined by the matching phrases introduced by the users. The system was evaluated by 15 patients with non-suicidal psychiatric disorders, who considered it to be appropriate for strengthening mental health. They also believed the platform would aid in the development of awareness, freedom of speech, and new insights. Laura is another RA that has shown effectiveness in administering informational and emotional social supports to older adults by delivering interventions and engaging in social and empathetic conversations with the user [45].

Ly et al. [46] investigated the effect and adherence to CBT for mental health delivery solutions provided by a fully integrated mobile app-based CA. This CA used the dialogue interaction function to have a chat with users about minor daily topics, and it responded based on the previous interactions. The chat conversations were pre-written by psychologists and centered on positive psychology thoughts, techniques, and activities. The investigators report that the CA may be strongly engaging while also promoting well-being and mitigating stress in a non-clinical setting, based on interviews with 14 individuals who used it. Morris and colleagues [47] developed a corpus-based CA that offers users instant empathetic support as well as automatic historical feedback to solve mental health problems such as stress, anxiety, and depression. The corpus included empathetic posts and associated responses. The CA compares the corpus to the user’s posts and extracts the best-ranked responses during the conversation periods. According to a randomized study of 1284 participants, 79.20% of them considered the CA to be satisfactory in terms of improving their emotional well-being.

#### 1.2.5. VAs for COVID-19

A systematic review [24] of the CAs developed for the COVID-19 pandemic indicates that, design-wise, simple rule-based CAs dominated this research domain, and targeted the following six public health use cases: risk assessment, information dissemination, surveillance, post-COVID-19 eligibility screening, distributed coordination, and vaccine scheduling. A few artificial intelligence-based CAs that were developed are described below.

Ouerhani et al. [25] proposed a cloud-based smartphone CA for anxiety assistance in post-quarantine circumstances during COVID-19, which aids in raising awareness of the outbreak’s true risk. Futhermore, the chatbot is able support users with management of stress both during and after infection. The CA was designed with the help of natural language processing (NLP) techniques to generate appropriate responses during the conversation with users. The researchers did not conduct any user evaluation of their CA, so design implications are not clear.

Ishii et al. [48] developed an empathy-driven embodied artificial intelligence-based CA, ERICA, that employed both verbal and non-verbal methods to help persons in self-quarantine feel less isolated. The authors compared ERICA with a web-based graphical user interface. They found that nonverbal exchange made by ERICA actually enhances the quality of the user experience during the screening session. The user feels that they are being empathized with and listened to. Their work highlights the importance incorporating nonverbal behaviors in dialog agents, especially for those in the mental health care domain.

Welch et al. [26] proposed an CA with the ability to show empathy and it could provide automatic counseling or persuasive interviewing to direct a person affected in COVID-19 toward stress reduction, and inspection of thoughts and emotions. This CA prompts users to provide open ended responses by asking questions structured around specific pandemic-related topics. The CA recognizes the topics being addressed (e.g., job, family), or emotions being felt (e.g., rage, anxiety), and then reacts with a reflective prompt. The researchers conducted a comparative evaluation study of their CA with a general purpose dialogue system, Woebot. Their findings indicate that users benefited from the reflective strategies used in their system and experienced meaningful interactions leading to reduced stress levels. This work points to the importance of using reflective strategies in dialog agents.

Battneni et al. [27] suggested a CA driven by artificial intelligence (AI) that can assist patients in remote areas by providing knowledge and COVID-19 virus updates. This CA determines the severity of infection by asking a series of predefined questions and then comparing user responses to a dataset. Further dialogs from the CA are created based on the feedback similarity score. Additionally, it may include therapy to assist people in recovering from psychological injuries sustained as a result of the pandemic’s trauma and anxiety. Woo et al. developed Akira [28], an AI-enabled CA, which is similar to Battneni et al.’s work [27]. The conversation model was built on a dataset with the help of NLP techniques. It has been trained with an accuracy of 90.6% using a deep neural network (DNN) model to communicate and react correctly in seven types of pandemic-related conversations, including mental health, cold and flu, medicines, drugs, and so on. Akira was evaluated by 57 participants, each of whom had a list of five questions to pose, and the user experience assessment showed that a greater testing dataset was necessary for improved results.

While these works explore CAs’ potential and suitability for delivering essential COVID-19-related health services, none investigated the true potential of RAs, i.e., their ability to maintain sustained socio-emotional relationships with patients and deliver health services according to patients’ unfolding needs. In the context of COVID-19, an RA can be more effective than a CA due to its potential to not only offer support and guidance but also provide companionship to patients during the self-isolation period. Moreover, RAs can remotely deliver essential health services, minimizing face-to-face interactions and preventing the transmission of infection.

In this paper, we present an RA that aims at supporting COVID-19 patients before, during and after the infection by providing relevant health services. We first outline our the study that began with the identification of six distinct actions (tasks) that the target RA must perform during non-life-threatening COVID-19 situations. We then designed trial conversations between a user and an RA to execute each task and refined them based on HCPs’ feedback. The final section discusses the potential of RAs in supporting patients during COVID-19 like pandemics, presents a novel methodology for uncovering design requirements of an RA being designed for novel health contexts, and discusses implications for designing an RA for COVID-19 pandemic.

## 2. Study 1: Identification of User Needs

The purpose of this study was to identify the kinds of help and support required by three different types of COVID-19 patients that could be provided by an RA. The three patient types or patient personas were identified in an earlier research [49] and they have been described in Table 1. Each persona represented a patient in a certain stage of COVID-19, i.e., suspecting infection (pre-infection), quarantining at home (during infection), and recovering after the infection (post-infection).

### 2.1. Methods and Materials

To uncover needs of COVID-19 patients in various stages of the disease, we chose to conduct a qualitative survey. With this approach, we were able to collect rich and open-ended input from a broad audience in a short time period. The survey consisted of three main parts. The first part was a brief overview of the study followed by an informed consent. In the second part, we collected participants’ demographical information such as age, gender, experience with smartphones, and infection status and severity. The final part of the survey was further divided into three sections. In each section, participants were introduced to a patient persona and then asked to draw on their own experiences to discuss how the persona could be supported. For example, in the section dedicated to the ‘Suspecting Infection’ persona, we first provided a description of the persona and, then, asked the participants to answer the following questions: (a) what do we need to know about the persona in this stage of the disease? (b) what kind of support does the persona need? (c) what kinds of problems is the persona likely to face? A similar procedure was followed for each persona.

The study was approved by the institutional review board of our home institution. We recruited participants by posting the survey on various social media platforms such as Facebook, Savvy, etc. The inclusion criteria for participation were: (a) at least 18 years old, and (b) currently or previously infected with COVID-19, and they were stated in the first part of the survey. The participants had to confirm that they meet the stated criteria in order to enter the actual survey. During the survey, the participants could skip any survey question they did not want to answer. Moreover, they could discontinue and quit at any time. The survey took approximately 30 to 45 min to complete and no one received any compensation for their participation. We kept the survey open for two weeks to ensure we have recruited enough participants.

### 2.2. Participants

Thirty-three participants (female = 17) with an average age of 40.05 years (SD = 12.20) participated in the study. Everyone considered themselves to be an expert smartphone user. Everyone except three participants had used a computer. Eighteen described themselves as regular computer users. 17 participants were familiar with or had interacted with CAs or voice assistants. In terms of infection severity, 25 participants had experienced mild symptoms, one was asymptomatic, and the remaining had severe symptoms. 16 had already recovered from the infection and the remaining were still in the post-recovery phase at the time of the survey. Seven participants had stayed at a hospital for a few days due to their critical condition, but they were at home at the time of the survey. Four participants had underlying conditions: one was a cancer survivor, two had diabetes (Type-1 and Type-2), and one had supraventricular tachycardia (SVT or heart condition).

### 2.3. Data Analysis

The qualitative data collected from the survey included responses to the open-ended questions about understanding the patient’s problems and support needs during the infection. This data was analyzed by two researchers using thematic coding techniques (i.e., annotation, open coding, and memoing). Each persona was analyzed independently. The researchers first independently browsed through the survey responses and made notes of their first impressions. They then read and re-read the data and annotated relevant words, phrases, and sentences with codes. The goal was to find personas’ needs, participants’ suggestions for helping the persona and any personal stories that highlighted challenges of going through this stage. The researchers then met to compare their codes for each persona and resolve differences through a discussion process. After the codes were finalized, the codes that were the same or reflected similar concepts were clustered together into categories. Since the category labels turned out to be very actionable, we did not further generalize them into themes and instead assigned them to personas as shown in Table 2.

### 2.4. Results

Based on our analysis, we identified two distinct tasks for the ‘Suspecting Infection’ persona, three tasks for the ‘Quarantining at Home’ persona, and one common task for all personas, including the ‘Recovering after Infection’ persona. In Table 2, we have listed all the identified tasks and the number of codes that were related to each task. In the following subsections, we discuss these tasks in more detail.

#### 2.4.1. Suspecting Infection: Screening for Symptoms

Participants explained that before they were diagnosed with the infection, they had extreme anxiety about their symptoms. A common problem faced by everyone was a lack of knowledge about the symptoms that were indicative of the infection. Some participants recommended that Oli (persona) should visit the primary care provider or the nearest health facility to get screened for the infection.

“Go to a nearby hospital for a preliminary checkup.” (P11).

Other participants advised against going outdoors and recommended that Oli should stay indoors for personal and communal safety (preventing the spread of the infection). Some participants had read articles or found online resources to identify COVID-19 symptoms and learned techniques to control them (e.g., self-isolate, wear a mask, drink hot tea, steam, etc.). Many participants had concerns about the reliability and accuracy of the online resources. They, specifically, stated that they could only trust the government websites for obtaining such information.

“Self-isolate, assume it is covid rather than flu, get in touch with a relevant medical helpline and ask for a test or go online to the covid help page and do the same. Stay in her room if there are others in the house with her. Warn them. Wear a mask. Use antibacterial sanitizer on hands. Clean taps, door handles, etc. after use. Drink plenty of fluids, take Tylenol and mix boiled warm water with honey, lemon, and ginger to soothe the throat as warm helps, honey and ginger have antibacterial properties and it soothes the throat.” (P7).

Overall, participants recommendations for Oli suggested that patients appreciate access to reliable and accessible sources that can provide answers to queries related to the disease. Specifically, patients want to identify which symptoms are concerning and which ones are not. Moreover, they value knowing what types of actions can help them control concerning symptoms and prevent further health deterioration.

#### 2.4.2. Suspecting Infection: Seeking Testing Guidance

Participants recognized that testing would be essential if Oli’s symptoms did not subside or became worse over time. They did not want Oli to visit a testing facility simply on a suspicion of infection, since many participants had been refused testing due to the absence of COVID-19 symptoms. Therefore, they wanted Oli to have enough information about their symptoms so they could decide whether the test was the right option for them.

Many participants had trouble locating the nearest COVID-19 testing facility but they had found it by calling up nearby pharmacies or their primary care providers, on a hunch.

“Call your primary care doctor. If you do not have one, call a CVS pharmacy or an immediate care facility. They can tell you where to go to be tested.” (P13).

While recounting their testing experiences, many participants stated that they had traveled for more than an hour simply to obtain a test, which was inconvenient for those who were experiencing exasperating conditions. Some participants recommended that Oli should opt for a home test but not everyone was aware of this alternate option.

This theme indicates that patients need guidance to determine the right time for a COVID-19 test. They may also need to be informed about the geographical locations of nearby testing centers. Moreover, patients need to be apprised of other options, i.e., home tests, in case they are too sick to travel alone, or there is no nearby facility.

#### 2.4.3. Quarantining at Home: Encouraging Healthy Behaviors

For the study participants, quarantining was about focusing on recovery and staying away from exhaustive activities. Participants explained that during the infection they felt physically weak and had limited energy to engage in regular activities.

“I mostly slept a lot. I could not concentrate for very long at a time, so I watched minimal television and did not read much at all. I played some games on my phone.” (P5, mild symptoms).

Other than trying to preserve their energy levels, participants also engaged in health-promoting activities to speed up recovery. They shared various health-promoting tips, which they had adopted during their own quarantine period, to ensure Oli was able to recover fully.

“Take vitamins, D, B12, C, Pain relief/ flu relief. Drink lots of fluids, warm drinks seem to help with the sore throat, a drink mixture with honey, lemon, and freshly grated ginger is good with boiled water. For loss of smell try re sensitizing olfactory nerves with smells like lavender and other natural products.” (P7).

Three participants (P6, P18, P19) explicitly mentioned that they had searched for specific strategies to control their symptoms on the Internet, e.g., “how to do breathing exercises?” (P6). Another participant (P9) mentioned that they had joined a Facebook help group to learn what they should expect during the infection from other infected individuals. There was an overall desire among participants to learn more about the infection progression and strategies to cope with concomitant challenges.

We also noticed that each participant’s suggestion for Oli was about adopting a specific type of strategy. For example, P5 and P10 suggested various ways to improve physical activity levels; P7, P9, and P21 proposed improving diet and focusing on herbal cures; P17, P12, and P13 recommended improving home ventilation and adopting holistic approaches to recovery, and so on. In other words, each participant was interested in adopting a certain type of behavior to become healthy.

This theme demonstrates that patients require informational and esteem social supports that align with their intrinsic values and strengths. This can consist of health promoting strategies that align with patients’ strengths and preferences. And also social supports that preserve their dignity and allows them to make a difference in other people’s lives. While it is important to take these factors into consideration, it is also important to ensure that patients’ preferences and values are not in conflict with clinical recommendations.

#### 2.4.4. Quarantining at Home: Requiring Personal Assistance

Many participants were living with their families. These participants relied on their family members and friends to have their daily needs met, e.g., grocery shopping, regular meals, and medicines. They were also aware taking care of them was burdensome on their family members.

“Food, drinks to door, my family did everything in the house exile. I was isolating, my husband slept in a camp bed and worked from home. My kids are more or less grown up but all had to come back. Two came after we had had covid, two were at home during covid. I have been struggling with fatigue ever since.” (P5).

The participants who lived alone expressed the need for a personal assistant. Many were able to get help from their friends with outside chores but, inside their homes, they had to do everything by themselves. Given that the participants were experiencing low energy and morale due to the infection, it was challenging for them to do self-care and house work on their own.

The infection takes a toll on patient’s health and strength. They might be able to do a few tasks on their own, but the burden needs to be lowered. This theme suggests that the RA should be able to provide some type of tangible support (i.e., goods and services that can help patients meet their self-management needs).

#### 2.4.5. Quarantining at Home: Handling Emergencies

Emergencies were an inevitable part of the COVID-19 journey but not all participants had prepared themselves to handle emergencies. Those who had prepared themselves had sought their physicians’ advice, searched for answers online and / or consulted online support groups. These participants were also equipped with appropriate medical devices such as a pulse oximeter, a thermometer.

Most of the participants were not aware of what could go wrong. These participants contacted their physicians, nearest hospitals, or 911 when their symptoms aggravated and when they were scared and did not know what to do. The main advice from their physicians and hospitals was to call 911, in case of emergencies. One participant had a doctor who made a house visit to check his lungs when he could not breathe normally.

“I have SVT [heart condition] and I had a couple of bouts of tachycardia while I was sick. I called my doctor and they suggested I call 911 if my heart rate did not decrease in 10–15 min.” (P4).

Patients may not know that they need to have a plan to contend with emergencies while they are in quarantine. Moreover, depending on their health condition(s) and/or the severity of infection, an emergency plan may need to be personalized to each person’s needs. At the minimum, information about the infection and contact information about professional services should be a part of patients’ emergency plans. Moreover, participants need access to appropriate medical devices, such as an oximeter.

#### 2.4.6. All Personas: Promoting Mental Wellness

Reassurances and emotional support were needed at every stage of the infection. During the pre-diagnosis stage, participants were scared and anxious about what might happen to them if they received a positive diagnosis. They sought emotional support from their family members and informational support from various sources to while experiencing this disease phase.

“I was afraid and anxious and didn’t know why I wasn’t getting better.” (P9).

During the quarantine stage, the need for emotional support was constant. Participants indicated that they felt emotional and negative about their illnesses. Persistent fever and worsening symptoms did not give anyone the opportunity to feel better. They needed encouragement and emotional support to make sense of their conditions. Participants relied on their family members, reached out to social support groups in their communities, and/or joined online support groups to receive emotional support. Some also contacted therapists to learn strategies to cope with the stress and uncertainty of the infection.

“I needed someone to talk to via telephone or forum that could reassure me that the symptoms I was experiencing were normal for COVID-19 infection.” (P17).

Post-infection, the severely infected and hospitalized participants continued to feel the trauma of hospitalization and a near-death experience. Witnessing the pain and suffering of other patients and the chaotic environment of the hospital had a profound impact on participants’ mental health. It was common for participants to connect with other individuals to seek answer to their present problems. Many participants lived alone and, hence, did not have the support of their friends or family members while experiencing the post-infection symptoms (long COVID-19).

“Some doctors and family members don’t understand, look for support groups online, most are going through the same thing, and speaking with others, is helpful.” (P14).

During their recovery, participants desired to return to their pre-infection state. They were trying to re-engage with their daily living activities, such as cooking or playing games, but, most participants were still feeling the impact of the infection. With long COVID-19 symptoms, there was a danger of developing a negative outlook on their lives. Patients need social supports to cope with mental health issues for each stage of COVID-19, i.e., pre-diagnosis, during, and post-infection, but a support system may not be readily available to everyone. Patients may have preferences regarding how and what kinds of mental health support they need.

## 3. Study 2: Evaluation of Conversations

We designed six conversations to demonstrate how the RA would engage a user to accomplish the six main tasks identified in the previous study (Study 1). Since the success of the RA is dependent on the effectiveness (i.e., ability to accomplish the target task) of the conversations, the goal of this study was to improve the effectiveness of the designed conversations through feedback from the healthcare professionals (HCPs).

### 3.1. Methods and Materials

We used a qualitative survey approach for this study to ensure we can obtain rich and open-ended feedback on the proposed conversations. To recruit participants, we emailed the survey to several mailing lists aimed at HCPs. Participation was voluntary and no identifying information was collected. Participants took 45–60 min to complete the survey.

**Figure 1:**
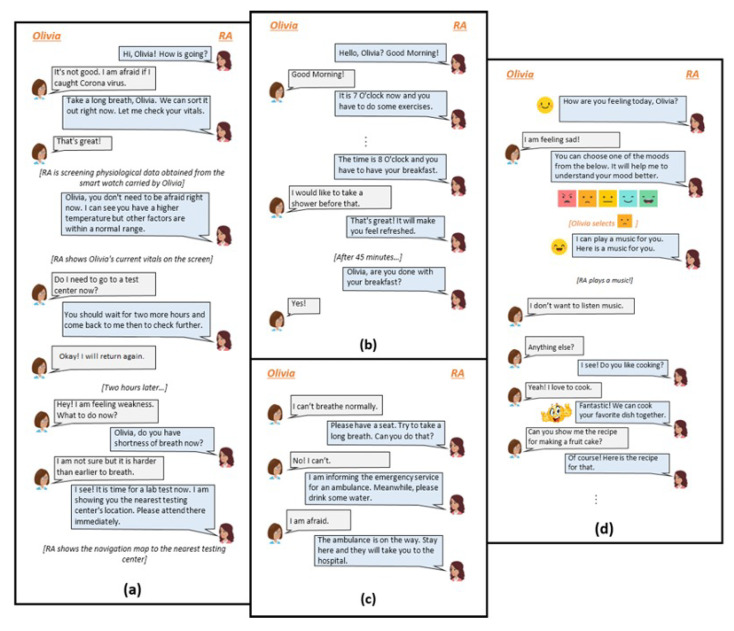
Snippets of designed conversations where the interaction between Olivia and the RA regarding (**a**) guidance for COVID-19 testing when Oli is suspecting infection; (**b**) practicing healthy behaviors for fast recovery when Oli is isolating at home; (**c**) handling an emergency during Oli home isolation; (**d**) managing anxieties during the post-recovery period.

The survey consisted of four parts. In the first part, participants were provided a brief overview of the study followed by an informed consent. The second part dealt with the collection of demographic data (e.g., gender, age range, education level, occupation, etc.); no personal information such as name or address was collected. Participants were also asked about their COVID-19 infection history and their experience with serving COVID-19 patients. The third part consisted of four sections. In each section, participants were first asked to review one conversation between the user and the RA aimed at accomplishment of a specific task. For example, participants were first shown the conversation between and RA and Oli who is ‘Suspecting Infection’ (Figure 1a), and then asked to respond to a series of questions. The survey questions were open-ended, eliciting feedback on participants’ judgments about the accuracy, completeness, and possible advantages of each conversation. Additionally, participants were asked to recommend ways to improve the proposed conversations. The same procedure was used to validate each proposed conversation with the target audience. Participants’ feedback on the overall conversation model was collected in the final part of the survey.

### 3.2. Proposed Conversations

The sample conversations used in this study were created in an iterative fashion using a combination of brainstorming amongst the designer and referencing the background research. During the brainstorming sessions, we closely reviewed participants’ comments from Study 1 to conceive dialogues between a patient and the RA. The conversations were refined several times before we landed upon a stable set of conversations that were then evaluated in this study. Our overall goal was to improve these proposed conversations by seeking input from the HCPs.

In each conversation, the RA is shown to be conversing with Oli Smith who is representing the target patient persona. Oli is assumed to be an expert user of smartphones and other digital tools such as tablet computers and voice assistants. It is also assumed that Oli has no underlying health conditions. The purpose of each conversation is to help Oli accomplish a certain task or provide a certain health service to Oli in response to an identified need. It is assumed that the RA has an underlying understanding of Oli and it is familiar with their preferences. A few snippets from the conversations have been included in Figure 1.

Figure 1a illustrates conversations between Oli (Suspecting Infection) and the RA. In this conversation, Oli is not sure whether they have contracted the infection but they are anxious about their existing exasperating condition. They approach the RA to gain some clarify about their symptoms. The RA responds by retrieving their vitals via a smartwatch (Screening for Symptoms) and, based on the analysis of this data makes further recommendations. If the vitals are indicative of COVID-19, the RA provides directions to the nearest COVID-19 testing facility (Providing Testing Guidance). Otherwise, it encourages Oli to practice social distancing and other health promoting habits.

The second conversation (Figure 1b) demonstrates interactions between Oli and the RA while Oli is in self-isolation at home as a result of being tested positive for COVID-19 (Quarantining at Home). Here, the RA is encouraging Oli to practice healthy habits to speed up recovery and prevent further health declines (Encouraging Healthy Behaviors). The conversation snippet in Figure 1c demonstrates how the RA handles an emergency, i.e., Oli reporting a severe breathing problem to the RA (Handling Emergency Situations).

Figure 1d shows how the RA will help Oli, who has just recovered from the infection (Recovering after Infection), regulate her mental health issue (Promoting Mental Well-being), regain confidence, and recover from the trauma they had experienced during their hospital stay. Here the RA is assumed to be familiar with the source of Oli’s trauma.

### 3.3. Participants

The following criteria were used to determine the eligibility for participation: (a) age of at least 18 years, (b) working in a healthcare setting, (c) familiarity with COVID-19-related guidelines and health concerns, (d) ability to speak, write and understand English, and (e) familiarity with mHealth technology. Forty-three HCPs (female = 29) ended up completing the survey in its entirety. In terms of occupation, 18 HCPs were providers (primary care, specialist, etc.), 21 were health care workers (registered nurse, nurse assistant, physician assistant, therapist, paramedic, etc.), 2 were medical students, and the remaining 2 were clinical social workers. Participants were of various ages ranging from 20 years to 60 years, with mean age 30.94 years (SD = 11.01). In terms of COVID-19 infection and symptoms, 28 had never been infected, 7 had recovered from the mild infection, 4 had recovered from severe infection, and the rest were asymptomatic. 19 HCPs had experience caring for a COVID-19 patient and everyone was staying abreast with the recent developments in COVID-19 by the virtue of reading research articles, newspapers, following changes in work protocols, and working in the COVID-19 intensive care unit, etc.

### 3.4. Data Analysis

The qualitative data were analyzed by two researchers using the thematic analysis techniques using coding and pattern searching [50]. The researchers first independently browsed the survey data to jot down their initial observations of the patterns. The second reading was more thorough during which researchers coded certain words, phrases, sentences, and paragraphs. Following this analysis, the researchers then met to compare their codes. The conflicts were resolved through a discussion process and once the codes were finalized, they were categorized to generate themes.

### 3.5. Results

Irrespective of their prior experience in caring for the COVID-19 patients, HCPs were proficient in terms of making suggestions for improving the proposed conversations. All nineteen HCPs who had cared for the COVID-19 patients believed that the proposed conversations with improvements would be sufficient for helping the patients with the three disease stages explored in this study.

The thematic analysis resulted in fifty-seven codes related to improving the proposed conversations (Table 3). These codes were clustered into four major themes and twelve categories. A substantial (22) number of codes were about improving the system’s robustness, i.e., redesigning it to more accurately mimic the clinical protocols and patient-provider interactions. For example, HCPs wanted to improve system’s robustness by having it model clinical interactions, providing care based on patient-centered principles, and incorporating evidence-based mental health interventions. The HCPs thought that the users would be more willing to trust an RA that is able to meet users’ realistic expectations and facilitate connections with actual HCPs. They believed that socio-emotional support must be shown by the RA through demonstration of empathy, validation of users’ emotions, and incorporating peer support. The study participants also recommended that the RA should be able to engage patients in their care by conducting periodic mood and health assessments. They also thought that education and reminders can also increase patient’s engagement with self-care. In addition, HCPs believed that age, income, and language differences would have an impact on the RA’s widespread acceptance. Hence, they recommended that the proposed system should be validated with a diverse population.

#### 3.5.1. Model Clinical Protocols

Three participants recommended improving the RA’s screening mechanism and accuracy by incorporating additional questions and a triage process. Triage is a medical practice that is used for the prioritization of care when there is a lack of resources [51]. It first determines the severity of a condition and then recommends appropriate actions based on identified needs. The participants recommended that the RA should start the screening process by asking questions about the presence and severity of symptoms. This would help identify those patients for whom COVID-19 testing or medical care (e.g., shortness of breath, fever) is urgently needed. Next, patients who have had direct contact with other COVID-19 positive individuals should be identified. These patients may not report concerning symptoms but require laboratory tests. Finally, patients who report no concerning symptoms or contact should have their vital signs evaluated.

“... A lot of questions could have been asked initially before giving the reassurance that everything was okay because most of her vitals were okay. The high temperature should have been enough of a reason to test. Also, should have asked if she had been exposed to someone who was positive, if she has lost taste/smell, what symptoms she was having, etc.” (HCP-14, Screening for Symptoms).

This theme shows that HCPs recognize that an RA can screen patients for the COVID-19 infection. However, the screener must be accurate, comprehensive, and robust, and based on established medical practices. Additional devices such as an oximeter, a thermometer, a heart rate monitor, etc., may be required to achieve this goal. Hence, such devices must be easily accessible to patients, and/or patients have the capability to interface such devices with the RA.

#### 3.5.2. Provide Person-Centered Care

Even though the conversations demonstrated that the RA had been designed to provide person-centered care to the user, participants recommended that the personalization feature of the system should be further refined. They explained that patients’ personal preferences and interests change based on the severity of their condition. They recommended that the health information and self-management strategies recommended by the RA must be aligned with disease symptoms instead of only with patients’ preferences. The HCPs explained that the symptoms of depression and anxiety aggravate with the progression of the COVID-19, which directly impacts patients’ engagement with the tasks and activities they had once enjoyed.

“If someone is truly depressed or suffering from a mental disorder, they likely won’t have the motivation to do a complicated task like cooking, even if they enjoyed doing it before.” (HCP-12, Supporting Mental Well-being)

The codes belonging to this theme appeared with the highest frequency, indicating that taking a person-centered approach needs to be at the forefront of RA-based care. This theme also indicates that the RA needs to stay updated with patients’ current health status and update its recommendation algorithm accordingly.

#### 3.5.3. Integrate Diverse and Evidence-Based Mental Health Interventions

Participants appreciated that the system was sensitive to patients’ mental health needs, and it tried to address them by suggesting coping strategies inspired by patients’ values at every stage of COVID-19 disease. However, they stressed the importance of incorporating evidence-based mental health interventions such as cognitive behavioral therapy, positive psychology, etc.

“... have evidence-based interventions in place for mental health or behavioral issues for people who are having mild cases. The anxiety of isolation as well as the anxiety of not knowing how severe the disease process is going to be is intense. Fatigue can also cause intense depression. It’s important for these symptoms to be normalized with tried and tested methods.” (HCP-6, Supporting Mental Wellbeing).

Participants also recommended that the RA should include evidence-based educational content that help help patients cope with anxiety, depression, and other common mental health issues that are likely to manifest due to the infection.

“I’ve cared for many patients who are suffering from COVID at home alone, and something that they all crave is mental health support. This is essential, especially for the elderly and those living at home on their own. Perhaps the bot may suggest an anxiety scale 1-5 and depression scale 1-5 and suggest telehealth psychologists or social workers for support.” (HCP-11, Encouraging Healthy Behaviors).

Mental health interventions are very important for COVID-19 patients. There are multiple ways to incorporate and deliver these interventions using an RA. HCPs recommend flexibility (offering multiple modes) and compatibility (matching user’s needs) when delivering these interventions. Since COVID-19 patients encounter mental health issues at every disease stage, it is important to incorporate a diverse array of interventions to ensure patients’ long-term engagement with the RA.

#### 3.5.4. Validate Patient’s Feelings

Participants also emphasized the importance of validating patients’ fears and concerns whenever they expressed their anxieties to the RA or whenever the RA detected changes in the patients’ mental health status through surveys.

“Telling someone not to be afraid when they are afraid is useless. Validate them and perhaps guide them through techniques to lower anxiety.” (HCP-5, Handling Emergency Situations).

“The RA should acknowledge that this is a tough illness and more will be coming down the pike. And, it should encourage the patients to learn to manage themselves over time.” (HCP-35, Handling Emergency Situations).

There are specific and sensitive approaches to communicating with those who are recovering from a challenging disease such as COVID-19. HCPs advised that specific approaches must be adopted in conversations to support the building of patients’ trust in the system.

#### 3.5.5. Set Realistic Expectations

Participants pointed out that the RA should not make a promote that it cannot deliver on. They believed that an important principle of building user’s trust in the RA should be about making sure that the user is not disappointed with the services offered by the system.

“Do not promise people they will be okay. Offer reassurance but don’t lie. Better to say, ‘you will get the best care possible’.” (HCP-10, Handling Emergency Situations).

Participants advocated for the RA to be autonomous in recognizing critical conditions and seeking emergency assistance, i.e., circumstances in which the patient may be unable to communicate and ask for help. They believed that RA’s ability to act autonomously is a realistic expectation, which users should be able to bank on in times of need.

“In some cases, patients can not respond and the guide should act independently to contact emergency services.” (HCP-12, Handling Emergency Situations).

The HCPs’ comments suggest that a clear understanding of the system’s capabilities has the potential to increase users’ trust in the system and make it more appealing to use. Moreover, the system must make its capabilities and services as transparent to the patient as possible.

#### 3.5.6. Facilitate Professional Connections

Participants suggested that besides designing the RA to provide mental health service, the RA should also allow patients to schedule remote consultation with human psychologists. Specifically, participants thought that this would be valuable for patients recovering from the infection and for those facing emergencies at home. They also recommended that the system should allow patients to connect with their primary or urgent care providers, in case they require consultation.

“Connect to a physician too if patient too afraid.” (HCP-7, Handling Emergency Situations)

HCPs believe that patients would feel more comfortable using a system that puts them in the vicinity of HCPs. In addition, such a system is likely to improve patient’s belief that the system can help them in emergencies. However, this suggestion conflicts with the original aim of the system, which is to minimize patients’ reliance on traditional healthcare facilities. If professional support is to be incorporated into the system, then it must be offered as the last resort after the users have exhausted all the resources offered by the system itself.

#### 3.5.7. Incorporate Peer Support

Participants unanimously agreed that the RA should allow patients to communicate with their loved ones. They believed that connection with their peers could be a very valuable for addressing patients’ mental health needs during the quarantine and the recovery periods.

“The requirement of physical and social distancing means we are no longer social beings. This has led to a lot of issues with isolation. When we are sick, we want to alleviate the burden of disease on our loved ones. The patients should be engaging their contacts 1-2 times a day to report their symptoms are okay.” (HCP-38, Encouraging Healthy Habits).

“Communication with loved ones may help in reducing PTSD. Inclusion of this feature is highly appreciated.” (HCP-9, Supporting Mental Well-being).

Moreover, participants thought that giving patients the ability to share their disease and recovery experiences with other COVID-19 positive patients would be an important step in improving patients’ mental health when they are scared.

“Communication should be encouraged among people who had COVID-19 to share their experiences.” (HCP-12, Encouraging Healthy Behaviors and Supporting Mental Well-being).

The HCPs believe that peer and family support is essential for improving the mental well-being of patients. HCPs encouraged that the patients not only share their previous experiences (related to COVID-19) with each other but also build new experiences together (e.g., by doing a psychological intervention session together). The RA should be able to create social network support and encourage the adoption of health-promoting behaviors in patient groups.

#### 3.5.8. Conduct Periodic Assessments

There was a consensus among the participants that the RA should be able to conduct periodic assessments of patients’ mental and physical health. This was considered necessary at all stages of COVID-19. HCP-2 recommended that the RA should periodically screen the patients for COVID-19 symptoms during the pre-diagnosis stage, regardless of whether the previous screening attempt resulted in a recommendation for testing or not. In both cases, the RA should ensure that the patient has received appropriate care and is not in any danger.

“Ask about more frequently symptoms, check in within 1 h vs. 2 h when patients are suspecting infection.” (HCP-2, Screening for Symptoms).

Participants suggested that during quarantine and post-infection periods, the RA should frequently be asking the patient about their health status, asking them to focus on their symptoms, monitoring their vital signs, and surveying them about the severity and presence of symptoms. The goal of these frequent check-ins is to figure out the care required by the patient and to provide them socio-emotional support. Another goal is to ensure that the patients receive care before their illness becomes too severe.

HCPs’ proposals clarify that periodic assessments by the RA would be beneficial for patients through all stages of the COVID-19 disease. Both passive (with the help of connected medical devices such as a pulse oximeter, a blood pressure, and a heart rate monitor) and active check-ins methods (i.e., surveys) were supported by the HCPs with the belief that having this would increase patient’s engagement with the system. However, it is also possible that constantly requiring feedback will increase patient fatigue and burden, causing them to abandon the use of the system altogether. Therefore, it is important to understand and adjust the frequency of these check-ins according to user’s response.

#### 3.5.9. Inform, Motivate, Remind

Participants suggested that the RA should provide accurate and up-to-date information about the pandemic. They recommended that the RA should deliver various kinds of educational content to the user. For example, they recommended that the RA should educate the user about how to manage the disease, such as learning to identify the alarming symptoms, how to do breathing/lung exercises to prevent exasperating conditions, encouraging patients to eat healthily and stay hydrated, and encouraging them to perform physical activities.

“Even if patients have normal vital signs and guidance is subsequently given for testing, it would conclude with an educational message with warning/alarm symptoms to look out for. If they feel bad, then do a critical assessment of not only your symptoms but also vital signs.” (HCP-6, Screening for Symptoms and Providing Testing Guidance).

“Patients should be taught the risk of not taking preventative measures. If they are not coughing, not moving, not taking deep breaths, then they are at risk of secondary infection.” (HCP-28, Encouraging Healthy Habits).

“Every hour, on the hour, remind them to get up and walk through the house for no good reason other than just to move. Motivating them is important.” (HCP-32, Encouraging Healthy Habits).

Participants indicated that the presentation of the educational content is important as it can have an influence on a patient’s ability to comprehend it and implement it in their lives. They thought that multimedia content would appeal to a wider audience.

“Including diagrams and images of COVID-19 test procedures and adding some helpline number.” (HCP-3, Screening for Symptoms and Providing Testing Guidance).

Health education and informational social support are important in the self- management of any disease. This theme suggests that HCPs believed that the RA has the potential to educate patients. It is important to understand how information is being presented to the users, since this can have a huge impact on a patient’s ability to understand and implement what is being presented to them.

#### 3.5.10. Accommodate Diverse Patients

The HCPs suggested that three factors (i.e., age, income, and language) should be considered in the design of the RA. Older adults, for example, usually have specific needs and limitations, which have an impact on their abilities to interact with the digital technology. For example, they might have hearing and visual impairment that can impact their ability to effectively use the RA.

The HCPs also stressed the importance of accommodating low-income patients because they felt that some features of the RA may pose a burden on them. For example, in the original conversations, the RA offers to order breakfast from a nearby café. The HCP pointed out that this might pose a burden on the patients. Moreover, income level also has an implication on what input and output devices the RA should be interfacing with, since low-income users may not be able to afford expensive external devices. However, acquiring income information from the user may be awkward and indirect methods could be used to obtain this data from the user.

Moving forward, it will be important to assess the feasibility of the proposed RA with different patient populations.

## 4. Discussion

The goal of this research was to understand how might we design an RA that could deliver COVID-19-related health services to patients outside the traditional healthcare facilities, ensure the safety of healthcare workers and mitigate virus transmission. In this paper, we have reported two studies that incrementally helped us identify tasks and characteristics of the target RA by building on our earlier work. The first study presented in this manuscript helped us determine specific tasks or health services for the target RA. The second study helped us decide how an RA should perform these tasks and how it should interact with the user while performing these tasks. Below, we present the following methodological and design implications of our work.

### 4.1. Methodological Implications

We used vignettes in both studies to investigate how might we design the target intervention. A vignette is defined as a “short story about hypothetical characters in specified circumstances, to whose situation the interviewee is invited to respond” [52]. Using a vignette in a research study allows participants to explore a particular situation from the point of view of the third person in the vignette. The purpose of using vignettes in Study 1 was to make it easier for participants to talk about their experiences with the disease. It is possible that talking directly about their infection experiences would have been a very traumatizing for the participants. The vignettes were meant to give participants the flexibility and control over when and how they wanted to use their own experiences to provide suggestions for the patient persona.

The purpose of using vignettes in Study 2 was to elicit HCP’s attitudes and beliefs about healthcare services that were being proposed by the target RA for the COVID-19 patients. The vignettes allowed HCPs to not only visualize the system’s capabilities but also gave them the power to modify it to reflect clinical values. Since we are proposing an innovative technology, we did not expect HCPs to have full knowledge of the strengths and weaknesses of an RA-based intervention. Hence, it would have been challenging for participants to imagine the conversations between the RA and the patient. We developed a separate vignette for each identified task to ensure we can receive a comprehensive feedback. Our vignettes (template conversations) made it easier for participants to imagine what is possible and how to make things better by tapping into their expertise and experiences.

Overall, this research demonstrates how the use of vignettes can lower the cognitive burden on research participants and support the exploration of design considerations for an innovative healthcare technology in unexplored contexts.

### 4.2. Design Implications

We found six healthcare services for COVID-19 patients: checking and controlling symptoms, providing testing guidance, supporting mental well-being, personal assistance, handling emergency situations, and encouraging healthy habits. We can find examples of RA in the literature that can handle these scenarios for other diseases. We found that our work echoes and extends the earlier findings in many ways. For example, Lucas et al. [19] and Zalake et al. [20] have developed RAs to screen patients for various conditions. However, their designs used structured approaches, i.e., a pre-defined questionnaire to determine patient status. Our work echoes this suggestion but, in addition, we suggest extending RA’s capabilities by allowing it to autonomously communicate with non-human actors to complete the screening process. Moreover, we suggest that the RA should be able to maintain a long-term relationship by periodically assessing symptoms and comparing them over time.

Designing the RA to build user’s trust in the system is supported by existing research, which suggests that RA’s conversations must allow users to experience quality interactions and build trust in RA’s capabilities [53,54,55]. Our research adds the following design recommendations around building users’ trust in health services-providing RAs. For example, participant HCPs suggested that an RA should avoid setting unrealistic expectations about its capabilities in the users. In essence, the HCPs wanted the RA to mimic HCPs’ knowledge, professionalism, and accuracy. The patients, on the other hand, suggested that they would value a knowledgeable companion during their journey through a disease as opposed to an entity that can only answer their queries or provide emotional support.

Earlier work in healthy habit engagement by Olafsson et al. [21] recommends routinely resetting engagement activities based on the user’s humor. While our work supports this suggestion, it also stresses the importance of personalized interventions and using the knowledge and information about patients to help them beyond their immediate needs. We recommend building conversations around the mutual participation model [56], which emphasizes an equal partnership between the HCP and the patient. The HCP’s responsibility is to evoke patients’ goals to design treatments in support of those goals. The patient, in turn, must share their life experiences and goals with HCPs so treatments can be designed according to the patient’s desires. Hence, RAs must strive to engage patients’ voices while demonstrating professionalism, knowledge, and accuracy in their conversations, so patients can develop trust and long-term relationships with these systems.

Although our knowledge about COVID-19 has evolved considerably since the time of these studies, most of the principles reported in this manuscript reflect the characteristics of patient-provider relationships and the general nature of care. As a result, these principles have broader application beyond the disease discussed. Nonetheless, there is one principle which might be more specific to the pandemic. That is, the HCPs suggested incorporating peers and professional services within the RA to support the mental well-being of patients. While earlier work such as Gaffeney et al. [44] recommend delivering cognitive therapy to PTSD patients using textual interactions without help from the HCPs. The overall goal of the target RA is to act as a replacement for HCPs and, possibly, peers. It is possible that due to the uncertainty at the time of the study, the HCPs felt that human presence was necessary. It will be interesting to test the validity of this principle when our knowledge of COVID-19 is more established.

The goal of this research was requirements analysis, which is necessary for any technical product in the early stages of development. The next step is the implementation of the proposed RA to validate it with the potential users. These days large language models are being used to enable natural conversations with the user [57]. These models are able to learn about text syntax and semantics by using self-supervised learning on huge quantities of text data. This allows them to generate conversation using minimum context. We recommend that large language models for RAs should be based on large quantities of text related to patient-provider relationships and bedside manners. The proposed design principles for RA can then fed be into these models to provide context for conversation generation. One consideration is that even though large language models demonstrate excellent text generation, the generated language comes from a probabilistic model, which can have an impact on the quality of the conversation. To guide this process, we have provided example conversations that demonstrate how the identified design principles can guide the conversation between the user and the RA (Refer to Table A1, Table A2, Table A3, Table A4, Table A5 and Table A6 in the Appendix A).

According to our findings, the RA must be able to support socio-emotional needs of the users by incorporating peer support. We can explore whether the RA can be designed to act like a peer, if it will not be possible to incorporate peers within the RA. Since RA research has explored physical appearances [39], or conversational styles of RAs [21], building a peer persona within an RA could involve investigating these two design aspects. Nonetheless, it is important to investigate how socio-emotional relationships between the user and the RA can be enhanced. The recent developmental psychology research has found that touch can help convey emotions, build interpersonal relationships and impact compliance in certain situations [58]. The future RA can be designed to interface with external haptic devices to convey sensation of touch. Furthermore, according to the socio-emotional selectivity theory [59], individual’s social goals are linked to their perception of time. When time is perceived as unlimited, knowledge-related goals are prioritized. In contrast, when time is perceived as limited, users prefer to pursue emotional goals. This has implications for user’s interaction with the RA. Additional psychology theories can be explored to further refine socio-emotional relationships between the RA and the user. Moreover, given that the socio-emotional intelligence of existing large language models are vague, these socio-emotional principles may help improve the emotional intelligence of the text generated by them.

This research uncovers what tasks an RA should perform for patients while going through various stages of COVID-19 and how an RA should communicate with its users for performing those tasks. We recommend that a health services RA-user relationship be based on a mutual participation model to support a patient’s trust and engagement in the system.

## 5. Limitations

There are a few limitations of the studies that must be mentioned. The task identification study is characterized by an unbalanced ratio between participants with mild and severe symptoms. We acknowledge that input from more severe participants might have offered a more suitable structure for scenarios related to emergency handling and post-infection recovery. While individuals with severe symptoms were included in the study, their health issues, such as weakness or inability to concentrate, may have prohibited them from responding appropriately throughout the study. As a result, these individuals may not reflect the whole population of COVID-19 survivors. The findings in the present manuscript are limited by the small sample sizes used in the two studies. Moreover, the results of this study are based on the input of people with technical expertise. We are, hence, lacking input from people without any technical skills. Additionally, we did not limit recruitment to a specific geographical location, but, most of the study participants were from the United States. This can also have an impact on the generalizability of findings. The diversification of the study population and replication of the study is likely to lead to further discoveries and generation of knowledge.

Several challenges involved in designing conversations for an RA have not been considered in this research. One of them is identifying the relevant and appropriate number of questions to ask the user as the RA engages in patient’s care. Another is identifying relevant responses to the user queries. Handling voice-based interactions in a conversational model and understanding voice tone and frequency are crucial to delivering effective output, especially when it deals with health service delivery. Additionally, real-time dialog generation based on user responses and situations is believed to be essential for the success of RA as a healthcare intervention [60]. In such instances, machine learning and natural language processing applications are needed for constructing a robust conversation model [61]. We are exploring these concerns in our future studies.

## 6. Conclusions

An RA aimed at supporting the COVID-19 patients before, during and post infection should be able to perform the following six tasks, i.e., symptom screening, providing testing guidance, encouraging healthy habits, providing support during self-isolation, handling emergencies, and supporting mental wellness of patients. To successfully perform these tasks, the RA should be clinically robust, capable of building socio-emotional relationships, promoting patient’s trust in the system, encouraging patients to engage in their own care and be able to accommodate a diverse range of patients. These design principles may help improve the quality of conversations generated by large language models. Finally, as a design tool, vignettes are helpful for validating early conversation designs with a population that is unfamiliar with the strengths and limitations of RAs. Future research should validate the recommended principles and proposed conversations with a diverse population.

## Figures and Tables

**Table 1:** Patient Personas for Study 1. Oli Smith lives in United States, owns a smartphone and has no underlying health condition, is playing the role of the persona in each case.

Suspecting Infection	Quarantining at Home	Recovering after Infection
Oli has been suffering from fever, cough, and headache for many days. They are worried about being infected by the COVID-19 virus.	Oli has been experiencing mild COVID-19 symptoms. They do not require hospitalization at this time. They have been advised to self-isolate at home until their symptoms subside. They feel isolated and fear the unknown.	Oli has recently recovered from severe COVID-19 symptoms that required hospitalization and a week in the intensive care unit (ICU). They are back at home and wish to recover as soon as possible. But they are experiencing post-traumatic stress disorder (PTSD). They feel that people around do not understand their struggles.

**Table 2:** Thematic Analysis Results from Study 1.

Personas	Category	No. of Codes
Suspecting Infection	Screening for Symptoms	23
	Seeking Testing Guidance	15
	Promoting Mental Well-being	17
Quarantining at Home	Encouraging Healthy Habits	28
	Requiring Personal Assistance	20
	Handling Emergencies	18
	Promoting Mental Well-being	20
Recovering after Infection	Promoting Mental Wellness	25

**Table 3:** Thematic Analysis Results from Study 2.

Theme	Category	No. of Codes
Improve System Robustness	Model Clinical Protocols	3
	Provide Patient-Centered Care	5
	Integrate Evidence-Based Interventions	10
Build Socio-Emotional Relationships	Validate Patient’s Feelings	4
	Incorporate Peer Support	4
Build Trust in the System	Set Realistic Expectations	3
	Facilitate Professional Connections	5
Promote Patient Engagement	Conduct Periodic Assessments	6
	Inform, Educate, Remind	7
Accommodate Diverse Patients	Older Adults	4
	Multiple Languages	2
	Low Income	3

## Data Availability

The data set can be obtained by emailing the corresponding author.

## Appendix A

Below we present the updated, full conversations for the six main RA tasks. The conversations are designed according to the principles that were identified in the second study. The applied design principles are included in the square parenthesis.

**Table A1:** Screening for Symptoms.

#	Conversational Exchange
1	Oli: CORA, I think I might have the COVID-19 virus! Can you help me sort this out?
	RA: Yes Oli, I am here for you. I have been trained by the National Institute of Health
	to screen people for COVID-19, which will help you decide whether you should get
	a test or not. [Go to #3] [Validate Feelings, Trustworthy sources, Realistic Expectation]
	Or
	RA: Hello Oli! My contact tracing algorithm is showing that someone at the party you
	attended last week has been diagnosed with COVID-19. [Active Sensing]
	Oli: Oh no! I know I should not have gone there. What should I do? [Go to #2]
2	RA: I am here to help. Since you are risk due to diabetes, I want to screen you for
	COVID-19 using the algorithm developed by the National Institute of Health.
	[Reference Trustworthy Sources, Patient-Centered Care]
	Please answer my questions as accurately as possible. Should we begin?
	[Model Clinical Protocols]
	Oli: Yes! I am ready. [Skip to #6]
3	RA: Please answer my questions as accurately as possible. Should we begin?
	[Model Clinical Protocols]
	Oli: Yes! I am ready. [Go to #4]
4	RA: During the last 14 days, have you been in close contact with someone who was
	at that time or since then has been tested positive for COVID-19?
	Oli: No, there has not been anyone like that. [Go to #5].
	Or
	Oli: Yes, last week I visited my nephew who just informed me that he has been
	tested positive for COVID-19. [Skip to #6].
5	RA: Have you attended any social gatherings, or visited any crowded places in the
	past 14 days?
	Oli: I am not sure. [Go to #6].
	Or
	Oli: Yes, I was at a party last week and the host just informed me that someone
	at the event was tested positive. [Go to #6]
6	RA: What is your body temperature right now?
	Oli: The thermometer is showing more than 100.4 degrees Fahrenheit. [Go to #7].
	Or
	Oli: My body temperature is reading normal at 98.6 degrees Fahrenheit. [Go to #7].
7	RA: Alright, but I must complete the screening for an accurate determination. Are
	you experiencing any of the following symptoms right now?
	· Loss of taste and smell,
	· Coughs,
	· Headaches,
	· Nausea or vomiting
	…. [More symptoms …]
	Oli: I have a runny nose and headaches. [Go to #8].
	Or
	Oli: I do not have any of these symptoms. [Skip to #9].
8	RA: Based on the screening results and your medical history, you may have
	the infection. It is recommended that you take the COVID-19 test immediately.
	Meanwhile, maintain optimal social distance and wear a face mask. I will check
	back with you in an hour. Let me know if anything comes up in between.
	[End] [Information and Education]

**Table A2:** Testing Guidance.

#	Conversational Exchange
9	RA: Oli, you do not have COVID-19 symptoms but you may still have the virus.
	Over the next two weeks, I will be checking back with you every day since the
	virus’ incubation period is 2-14 days. If you experience shortness of breath,
	coughing, high temperature, aches, [other symptoms], contact your primary care
	provider right away. Meanwhile, please practice social distancing and wear a face
	mask. You can also order a home test kit from the NIH-recommended DDNest Labs
	to self-test for the virus. If you do a self-test and find yourself positive, please
	inform me right away. [End] [Periodic Assessments, Information and Education]

**Table A3:** Screening for Symptoms.

#	Conversational Exchange
1	Oli: Hey! I am not sure where to go for my COVID-19 test?
	RA: No problem, Oli! I can guide you. The nearest testing facility is
	2.5 miles away from your current location. [Go to #2]
2	Oli: What is the address?
	RA: DDNest Pharmacy is located at 301 East Lewis Street, Lafayette,
	Louisiana 70504. [Go to #3]
3	Oli: Do I need an appointment for the test?
	RA: They are open to receiving walk-ins. Just tell them that you are there for
	a test. Use the drive-thru facility because you may be infected. [Go to #4]
4	Oli: I want to go. But I am not feeling so good. Can you remind me in 15 min?
	RA: No problem! I will ping you in 15 min. [Frequent Check-ins] [Go to #5]
5	[After 15 min]
	RA: Oli! Are you ready for the test now?
	Oli: Yes, please provide me driving directions to the pharmacy [Skip to #8].
	Or
	Oli: I am feeling better but I am afraid I will become too sick during my drive.
	[Go to #6]
6	RA: I understand your fears. If something happens on the way, I can call someone
	for you. Do you have anyone in mind? [Validate feelings]
	Oli: Yes, can you call my mother. Her number is 123-456-7890. [Go to #7]
7	RA: Alright! Just say “Call Mom” when you feel the need to talk to her and
	I will connect you to her. I will also keep you company during your drive.
	[Peer Support, Frequent Check-ins]
	Oli: I feel assured now and am ready to go. I want the driving directions.
	[Go to #8]
8	RA: Sure! The navigation map is on your phone screen now. I am also here for
	you. [Go to #9]
9	[15 min after arrival to the destination]
	RA: Have you completed the test yet?
	Oli: Yes, they have taken my sample. The result will be available within the next
	two days. [Go to #10]
10	RA: When you receive the test results, please inform me right away. I will check
	back with you to make sure your symptoms are not worsening. Meanwhile, maintain
	optimal social distance and wear a face mask. Let me know if I can be of further
	assistance. [End] [Frequent Check-ins, Information and Education]

**Table A4:** Encouraging Healthy Behaviors.

#	Conversational Exchange
1	RA: Good morning, Oli!
	Oli: Good morning!
2	RA: It is 7 O’clock now. You need to do some exercise. [Remind]
	Oli: I do not feel like exercising right now.
3	RA: Oli, You must do some physical activity to speed up your recovery and
	to maintain your energy levels. It will also boost your mood. [Educate]
	Oli: Okay, but I do not have the energy. What should I do?
4	RA: Not a problem! I can be your exercise coach. Just sit up for now and
	breathe deeply for a minute. I will time you.
	Oli: Okay! I am sitting now. Can you start timing?
5	RA: Yes, let’s start!
	[After one minute]
	RA: Very good, Oli! Your one minute is over. Now whenever you are ready,
	stand up and walk around the room for 5 min. Let me know when you are
	ready so I can time you. [Inform]
	Oli: Okay, I am ready.
6	RA: Timer started!
	[After 5 min]
	RA: Excellent, Oli, Now let’s do some lung exercises. Can you see the exercise
	animation on the screen? Just follow what they are doing. [Educate]
	Oli: Wow! I will get on this right away.

**Table A5:** Providing Personal Assistance.

#	Conversational Exchange
1	Oli: I am hungry, but I do not have the energy to prepare my breakfast.
	RA: Do you want me to order your favorite breakfast from Sunshine Cafe?
2	Oli: That will be nice, but I am afraid that will be too expensive for me.
	Can you call my neighbor? They said they would leave food for me in the patio,
	if I called them.
	RA: Sure! [Incorporate Peer Support]
	[Oli talks to the neighbor on the phone]
3	RA: Oli, A shower may make you feel refreshed.
	Oli: Yeah! It’s a nice idea. I will shower now. Meanwhile, the food will arrive.
4	[… after 30 min …]
	RA: Oli, did your food arrive?
	Oli: Yes, it did. I am almost done with my breakfast.
5	[… after 15 min …]
	RA: Don’t forget to take Levothyroxine when you are done!
	Oli: Thanks for the reminder! This infection is making me forget everything!
	[Remind]
6	RA: Also, please don’t forget to take a Vitamin C caplet.
	Oli: Of course! I love them. [Remind]

**Table A6:** Handling Emergencies.

#	Conversational Exchange
1	Oli: I am having a hard time breathing!
	RA: Oli, Try to relax. Can you take a deep breath?
2	Oli: I can but I am feeling congestion in my chest.
	RA: I understand! I will try my best to help you out. [Validate Feelings]
3	RA: Do you have a pulse oximeter?
	Oli: Yes! I have one.
4	RA: Please put it on the end of your finger and tell me the reading.
	Oli: Okay! The oximeter is showing 80%.
5	RA: Oli, your oxygen level is low. A doctor must perform a more formal
	assessment. I am calling 911 to get help. Until someone arrives, please
	lie down on your belly as it will help you breathe better. [Provide
	Professional Connections]
	Oli: Okay!
6	[After a few minutes]
	RA: How are you feeling now?
	Oli: It’s better than before. But, I hope the ambulance arrives soon.
7	RA: They said they are coming in 15 min, provided road conditions
	are normal.
	Oli: Okay, I think I will be fine until then.
